# NS1 Recombinant Proteins Are Efficiently Produced in *Pichia pastoris* and Have Great Potential for Use in Diagnostic Kits for Dengue Virus Infections

**DOI:** 10.3390/diagnostics10060379

**Published:** 2020-06-06

**Authors:** Mariana Fonseca Xisto, John Willians Oliveira Prates, Ingrid Marques Dias, Roberto Sousa Dias, Cynthia Canedo da Silva, Sérgio Oliveira de Paula

**Affiliations:** 1 Department of General Biology, Federal University of Viçosa, Viçosa, Minas Gerais 36570-900, Brazil; marianaxisto@hotmail.com (M.F.X.); ingridmdias@hotmail.com (I.M.D.); rosousa318@gmail.com (R.S.D.); 2 Department of Microbiology, Federal University of Viçosa, Viçosa, Minas Gerais 36570-900, Brazil; john_prates@hotmail.com (J.W.O.P.); ccanedosilva@gmail.com (C.C.d.S.)

**Keywords:** dengue, NS1 protein, *Pichia pastoris*, diagnosis

## Abstract

Dengue is one of the major diseases causing global public health concerns. Despite technological advances in vaccine production against all its serotypes, it is estimated that the dengue virus is responsible for approximately 390 million infections per year. Laboratory diagnosis has been the key point for the correct treatment and prevention of this disease. Currently, the limiting factor in the manufacture of dengue diagnostic kits is the large-scale production of the non-structural 1 (NS1) antigen used in the capture of the antibody present in the infected patients’ serum. In this work, we demonstrate the production of the non-structural 1 protein of dengue virus (DENV) serotypes 1–4 (NS1-DENV1, NS1-DENV2, NS1-DENV3, and NS1-DENV4) in the methylotrophic yeast *Pichia pastoris* KM71H. Secreted recombinant protein was purified by affinity chromatography and characterized by SDS-PAGE and ELISA. The objectives of this study were achieved, and the results showed that *P. pastoris* is a good heterologous host and worked well in the production of NS1DENV 1–4 recombinant proteins. Easy to grow and quick to obtain, this yeast secreted ready-to-use proteins, with a final yield estimated at 2.8–4.6 milligrams per liter of culture. We reached 85–91% sensitivity and 91–93% specificity using IgM as a target, and for anti-dengue IgG, 83–87% sensitivity and 81–93% specificity were achieved. In this work, we conclude that the NS1 recombinant proteins are efficiently produced in *P. pastoris* and have great potential for use in diagnostic kits for dengue virus infections. The transformed yeast obtained can be used for production in industrial-scale bioreactors.

## 1. Introduction

Dengue is a systemic infectious disease, globally distributed by establishing cycles of endemic and epidemic transmission [1,2]. It is caused by viruses of the *Flaviviridae* family, and is transmitted among humans in urban regions by mosquitoes belonging to the genus *Aedes* [3]. In some cases, the infection is not apparent, but can cause various clinical manifestations, from low fever to dengue shock syndrome (DSS) and dengue hemorrhagic fever (DHF), which can be fatal [4]. This virus has four antigenically different serotypes, dengue virus serotypes 1, 2, 3, and 4 (DENV1, DENV2, DENV3, and DENV4) [5], of which the serotype DENV2 is responsible for causing the most epidemics [6]. Immunity developed after infection by one of the four serotypes does not protect the patient from reinfection by another viral serotype, which may lead to severe clinical conditions of the disease [7]. Dengue is endemic in more than 120 countries, accounting for 3.9 billion people at risk of infection worldwide—three times more than the World Health Organization (WHO) has reported [8]. The manufacturers of the tetravalent vaccine Dengvaxia^®^ (Sanofi-Pasteur), released in 2015 [9], received recommendations from the WHO to carry out more tests due to the risk of complications to individuals who had never had the disease previously [10]. 

Dengue is a single-stranded RNA, icosahedral, enveloped virus. The genome encodes three structural proteins (capsid (C), envelope (E), and membrane (M)) and seven non-structural proteins (NS1, NS2A, NS2B, NS3, NS4A, NS4B, NS5) that are responsible for the virus genome replication. The replicative complex (formed by NS2A to NS5) is mounted on the endoplasmic reticulum (ER) membrane on the cytoplasm side [11,12,13]. 

The non-structural 1 (NS1) protein is the first translated protein and plays a key role in virus replication [14,15,16]. It is a glycoprotein with molecular mass ranging from 46 to 55 kDa, depending on the glycosylation pattern, and is composed of three structural domains: β-roll, wing, and β-ladder. In the dimeric form, it is associated with the ER membrane lipids, aiding viral genome replication. In the hexameric form, it is secreted by infected cells, and interacts with complementary components of the immune system, related to the immune evasion and disease pathogenesis [17,18,19,20]. NS1 is found in the serum of infected patients at the early stages of the disease, and is used as a biomarker for the early diagnosis of dengue [21,22]. It is highly immunogenic, inducing the production of IgM (acute phase) and IgG (convalescence phase) antibodies that are detected in the capture enzyme immunoassays [23].

Over the course of the past few years, dengue infections have led to particular attention in terms of public health, having spread and reaching regions where cases are often not reported and notified [24,25,26]. The WHO has indicated the classification of the severity of the disease and the warning signs for hospitalization of patients with more severe conditions [27]. However, it is not possible to depend only on the clinical manifestations, since many infections are asymptomatic, present nonspecific clinical signs, and need a differential diagnosis [28,29]. Therefore, a quick, accurate, and low-cost diagnosis is essential to confirm suspicions of cases of dengue fever, favoring disease prevention in regions of infection and treatment of infected patients, especially in countries where health care resources are limited and inaccessible [30,31,32].

The Special Program for Research and Training in Tropical Diseases, the WHO, and the Dengue Scientific Working Group have established research priorities to provide information and encourage developmental strategies for reversing the dengue epidemiological trend, as the current global epidemic has persisted for more than 10 years. Three main goals were set: reduce mortality by 50% and morbidity by 25% by the year 2020, and maintain focus on efforts and research [33,34]. 

Using yeast for the expression of eukaryotic proteins has been extensively explored because of its capacity for producing large quantities of heterologous proteins [35,36]. It is an organism which permits easy genetic manipulation, thus allowing optimization for expressing specific proteins. Many proteins of commercial and pharmaceutical interest are proteins related to human biological functions, and several have post-translational modifications that can best be performed on yeast expression systems rather than in bacterial expression systems. The advantages of using yeast are the rapid growth rate, reduced cost, and the fact that large-scale production can be performed using fermentation [37,38].

For decades, the methylotrophic yeast *Pichia pastoris* has been widely used for the expression of proteins with therapeutic and industrial uses [39]. It is able to grow in the presence of methanol as the unique carbon source and energy. Through genes related to methanol degradation (the AOX1 gene), biotechnology has developed strategies for the efficient production of recombinant proteins [40,41,42]. The advantage of *P. pastoris* is mainly due to the high secretory capacity of the produced proteins, related to the efficiency of the strong promoters [43,44,45].

In recent times, some research groups have published works expressing non-structural 1 (NS1) protein of Dengue in bacteria *Escherichia coli* as a tool for large-scale expression [46,47] and *Baculovirus* [48,49,50] as a tool for large-scale expression, and the correct fold and glycosylation in *Pichia pastoris* has already been described [51].This work, *Pichia pastoris* yeast was transformed genetically to produce non-structural protein 1 of the viruses DENV1, DENV2, DENV3, and DENV4. We used the strain KM71H—a mutant strain that has the advantage of growing in minimal medium, without the need of supplementation with essential amino acids as wild strains. Therefore, aiming for large-scale production, it becomes a good option for reducing fermentation costs. The recombinant proteins showed high antigenic potential, with the ability to be recognized by anti-dengue antibodies in positive serum samples, when used in enzyme-linked immunosorbent assays. The study is a step forward for the commercial-scale production of antigens to be used in serological tests for dengue diagnosis. 

## 2. Materials and Methods 

### 2.1. Strains and Vectors 

The strain used in this study was the methylotrophic yeast *Pichia pastoris* KM71H. This lineage has the enzyme alcohol oxygenase 1 (AOX1) gene deleted, so that only the alcohol enzyme oxygenase 2 (AOX2) gene is functional. It is a mutant strain (MutS) and shows reduced consumption of the methanol inducer as a carbon and energy source [40]. For secretory expression, the integrative plasmid pPICZαA (Invitrogen, Carlsbad, CA, USA) was used, which has the AOX1 promoter fused to the pre-pro-factor (encoding the signal peptide to the secretion pathway) and a Zeocin™ resistance marker for transforming yeast selection. *E. coli* TOP10F was used for cloning and replication of constructs.

### 2.2. P. pastoris Cloning

Gene sequences encoding NS1 proteins were synthesized and optimized for expression in yeast using cloning vector pUC57 (GenScript, Piscataway, NJ, USA). Each gene was inserted between two specific restriction sites independently in the pPICZαA vector: *EcoRI* and *NotI* (DENV1, 3 and 4) and *KpnI* and *NotI* (DENV2). Constructs (pPICZαA_NS1-DENV1–4) were linearized with *SacI* and used in *P. pastoris* KM71H transformation by electroporation. Transformants were selected on Zeocin™-resistant YPDS (1% yeast extract, 2% peptone, 2% d-glucose, and 18.2% sorbitol) at different antibiotic concentrations (100, 200, and 500 μg/mL), as described in the Easy Select *Pichia* Expression kit (Invitrogen). Genomic DNA of the transforming clones was extracted, according to Looke and Kristjuhan [52], with minor modifications, and insertion was confirmed by PCR using specific primers for AOX1: AOX1sense (5′gactggttccaattgacaagc3′) and AOX1antisense (5′gcaaatggcattctgacatcc3′).

### 2.3. Recombinant NS1-DENV1–4 Protein Production

PCR-confirmed transformants were pre-inoculated into 5 mL of YPD medium (1% yeast extract, 2% peptone, and 2% d-glucose) and maintained for 24 h at 30 °C while agitating at 200 rpm. Each culture was inoculated into 1 L of BMG minimal medium (1.34% YNB, 0.002% biotin, 1% glycerol, and 100 mM potassium phosphate, pH 6.0) and maintained for 72 h at 30 °C while agitating at 250 rpm until OD600 ≈ 20 was reached. Then, yeasts were recovered by centrifugation and solubilized in 500 mL of the BMM induction medium (1.34% YNB, 0.002% biotin, 100 mM potassium phosphate, pH 6.0, 1% methanol) supplemented with 1% casamino acids, which according to the work of Kaushik and Rohila [53] help in proteolysis reduction and favor recombinant protein secretion. Yeast cultures were maintained for 96 h at 20 °C under agitation (250 rpm), and at 12-h intervals, the media was supplemented with 0.5% (*v/v*) methanol. Finally, the cultures were centrifuged and the supernatant collected to purify the proteins.

### 2.4. NS1-DENV1–4 Purification

Supernatants containing the recombinant proteins (approximately 500 mL) were diluted in 500 mL of binding buffer with pH 7.4 (20 mM sodium phosphate, 500 mM NaCl, and 20 mM imidazole) and subjected to affinity chromatography. A 5 mL HisTrap^®^ Fast Flow Crude (GE HealthCare^TM^, Chicago, IL, USA) column, previously equilibrated with the binding buffer, was coupled to the AKTA purification system (GE HealthCare^TM^). Proteins were recovered using an elution buffer with pH 7.4 (20 mM sodium phosphate, 500 mM NaCl, and 400 mM imidazole), lyophilized and solubilized in 100 mM of Tris-HCl buffer, pH 8.8. Quantification was estimated by the BCA kit (Pierce Chemical Co., Rockford, IL, USA).

### 2.5. Recombinant Protein Characterization

After purification, NS1-DENV1–4 proteins were electrophoresed (SDS-PAGE 12%, stained with Coomassie Blue) and transferred to a nitrocellulose membrane. After transference, the immunolabeling was done. In order to do this, each membrane was blocked with PBS (10 mM phosphate, 137 mM NaCl, and 2.7 mM KCl) supplemented with 3% gelatin, and a pool of 3 polyclonal serum samples from patients infected with dengue virus was used as a primary antibody. Membranes were incubated for 24 h, and after this time were washed 5 times with PBS-Tween-20. Then, a secondary antibody with alkaline-phosphatase-conjugated anti-human IgM (Sigma, St. Louis, MI, USA) was added to the membranes for 2 h. After incubation, the membranes were revealed with BCIP/NBT (Sigma). For the labeling of glycoproteins, a reaction was performed using periodic acid-Schiff (PAS). NS1DENV1–4 proteins were electrophoresed (SDS-PAGE 12%) and transferred to nitrocellulose membrane. After the transfer, membranes were washed with 12% trichloroacetic acid for 5 min. The next steps were carried out at 4 °C in a dark room. Membranes were incubated with 0.5% periodic acid for 15 min, and washed 3× with 15% acetic acid for 5 min. Then, 50 mL of Schiff’s reagent was added and incubated for 30 min. Finally, 6 washes were performed with 7.5% acetic acid for 1 h, and the membranes were dried at room temperature.

### 2.6. Serum Samples

In this study, 192 serum samples from the Laboratório de Atenção à Saúde in the State of Rondônia (Laboratory for Attention to Health, LACEN/RO) and the Banco Central de Sangue of the State of Rondônia, Brazil (Central Blood Bank, FHEMERON/RO) were used. All sera were tested in accordance with protocols approved by the Central Blood Bank of the State of Rondônia, Brazil and all samples were kept anonymous, project identification FAPEMIG APQ-00412-18, date 01 April 2018.

Samples were previously confirmed as IgM-positive or IgG-positive for dengue by IgM MAC-ELISA (Pan-Bio, Brisbane, Australia) and Duo Capture ELISA Kit IgM and IgG (Sanofi, New Jersey, NJ, USA).

### 2.7. ELISA

The recombinant proteins NS1-DENV1, NS1-DENV2, NS1-DENV3, and NS1-DENV4 were used as coating antigens to sensitize 96-well high-binding ELISA plates (JetBiofil, Seoul, Korea) at 1 μg/well in a carbonate-bicarbonate buffer, pH 9.6, and incubated at 4 °C for 24 h. Patient serum samples were diluted to 1/100, added in duplicates to the plates, and incubated at 37 °C for 3 h. Subsequently, the plates were washed 5× with PBS, and 0.05% Tween-20 was added. Peroxidase-conjugated anti-human IgM (Sigma) and anti-human IgG (Sigma) secondary antibodies were added to the respective plates at 1/2500 dilution and incubated at 37 °C for 2 h. After incubation, the plates were washed 5 times with PBS, 0.05% Tween-20, and ABTS substrate (2,2′-azinobis (3-ethylbenzothiazoline-6-sulfonic acid) diammonium salt) (Sigma) were added, and they were incubated for 20 min at room temperature. After reaction blocking with H_2_SO_4_, the plates were read (OD450 nm) in a multi-channel spectrophotometer (MultiskanGo, Thermo Scientific, Waltham, MA, USA).

### 2.8. Statistics

Receiver operating characteristic (ROC) curves were analyzed to estimate the diagnostic cut-off, sensitivity and specificity (Prism7 Software, GraphPad, EUA). The unpaired *t*-test was used to assess the significance of the ELISA assays, a parametric test applied to two different samples, where both groups are independent.

## 3. Results

### 3.1. Pichia pastoris NS1-DENV1–4 Cloning 

**Figure 1:**
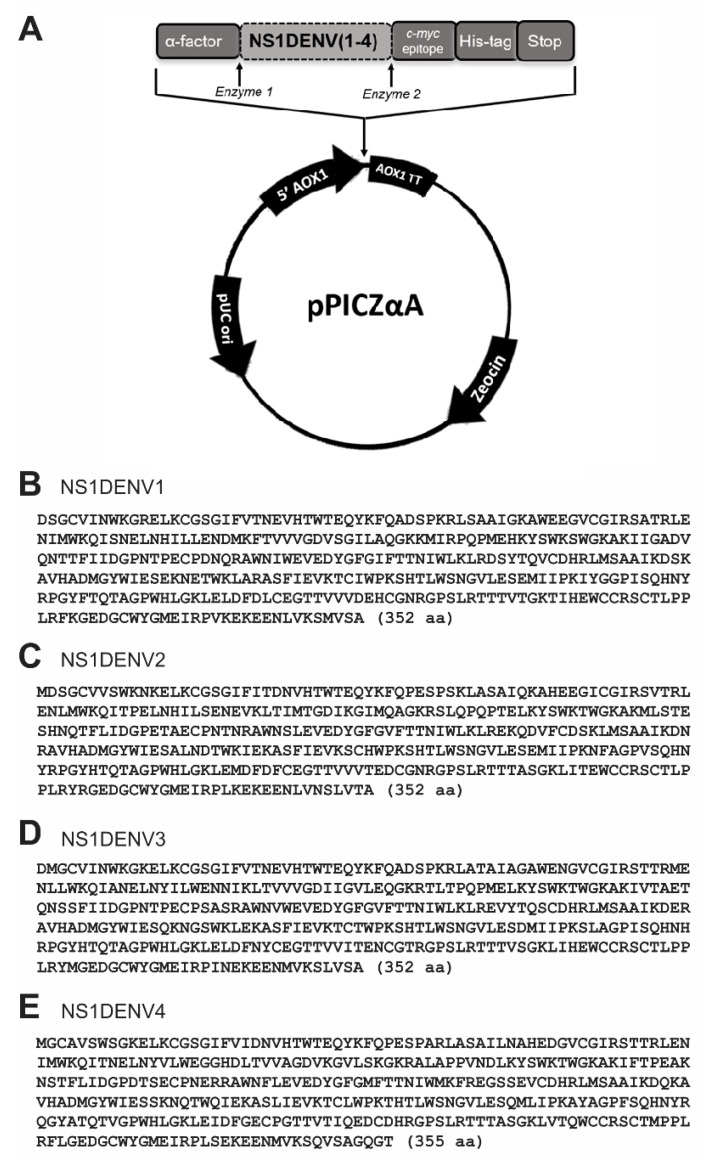
Construction of expression vectors pPICZαA_NS1DENV1–4. (**A**) Plasmid map: alcohol oxygenase 1 (AOX1) promoter with terminator region. Zeocin resistance gene. pUC ori for start replication in *E. coli*. Cloning cassette: α-factor secretion signal, NS1 gene, *c-myc* epitope (not used in this work), His-tag, stop codon and restriction enzyme sites; (**B**–**E**) Amino acid sequences that were codon-optimized for yeast expression of viral proteins (DENV1, DENV2, DENV3, and DENV4).

Four expression vectors were cloned from pPICZαA plasmid, with codon-optimized sequencing of the NS1 proteins from the DENV1–4 viruses (Figure 1A–E). Each construct yielded one protein, which has a polyhistidine tail (6× His) fused in the C-terminal portion for purification and detection.

**Figure 2:**
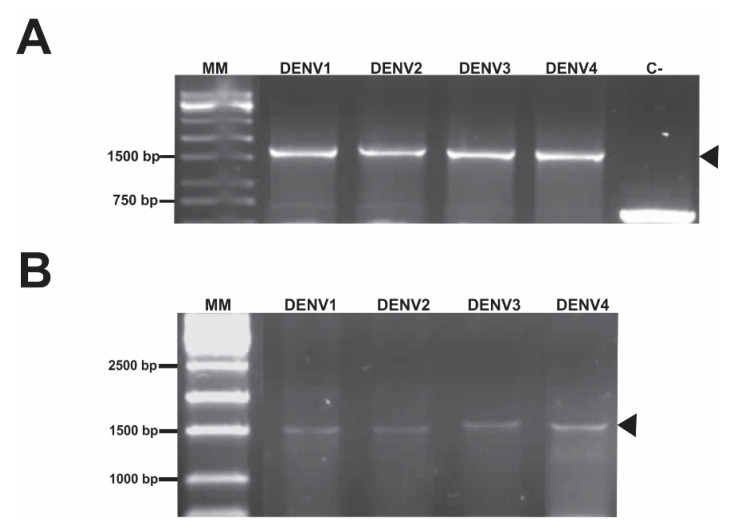
Confirmation of cloning and *Pichia pastoris* transformation by PCR. (**A**) Independent insertion of the NS1DENV1–4 genes into the vector pPICZαA. MM: molecular marker; DENV1–4: positive amplifications of DENV1–4 genes; C-: negative control (pPICZαA empty). (**B**) PCR of the genomic DNA of the recombinant yeasts positive to NS1DENV1–4.

Insertion of the genes into the expression vector pPICZαA_NS1DENV1–4 was confirmed by polymerase chain reaction (PCR), and the empty plasmid was used as a negative control (Figure 2A). Positive gene amplification from each recombinant clone corresponded to the amplicon generated by the AOX region (downstream and upstream) added to the gene encoding the protein, and negative amplification corresponded to the AOX region amplicon without the inserted gene.

For *P. pastoris* transformation, vectors were linearized with the *SacI* enzyme, and electroporation was done to obtain recombinant clones. DNA from the Zeocin^®^-resistant transformants was extracted and PCR was performed using specific primers for the AOX1 gene to correct cloning confirmation. Amplificons confirmed that each gene was independently integrated into the AOX1 locus of the *P. pastoris* KM71H host genome (Figure 2B).

### 3.2. Expression and Purification of Recombinant Proteins

**Figure 3:**
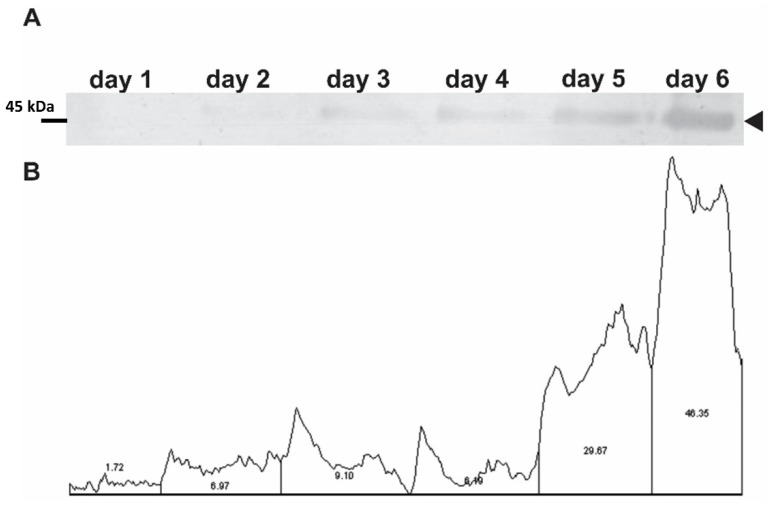
Quantitative analysis of expression in 6 days. (**A**) SDS-PAGE of the NS1DENV2 protein expressed on days 1 through 6; (**B**) Densitometry of the gel bands.

A quantitative characterization of the expression was done to determine which day the methanol-induced yeasts produced a peak of recombinant proteins. Samples were collected from the yeast culture medium from day 1 to day 6 during NS1DENV2 protein production, and it was defined by SDS-PAGE that the sixth day was ideal for protein purification (Figure 3). All four yeasts presented the same maximum expression profile on the sixth day. After induction and expression, the recombinant proteins (NS1DENV1, NS1DENV2, NS1DENV3, and NS1DENV4) were purified by nickel column affinity chromatography. The fractions that showed peak absorbance were collected, lyophilized, and quantified for yield calculation (Table 1).

**Figure 4:**
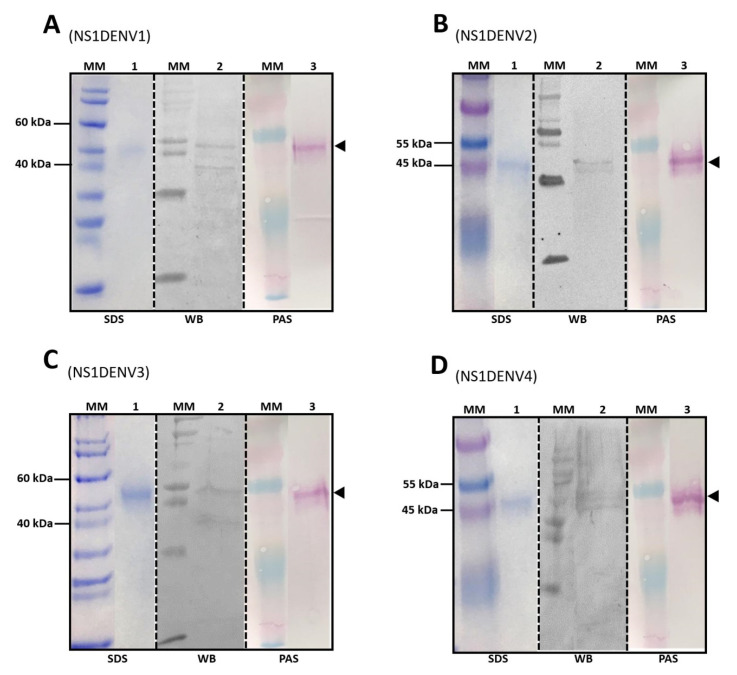
Molecular characterization of recombinant proteins. MM: protein molecular marker; (1) SDS-PAGE; (2) Western blot using anti-dengue positive serum pool; (3) Periodic acid-Schiff staining: (**A**) NS1DENV1, (**B**) NS1DENV2, (**C**) NS1DENV3, and (**D**) NS1DENV4. Arrowheads show the glycosylated form of the NS1 protein.

Purified fractions of the four recombinant NS1DENV1–4 proteins were evaluated in electrophoresis on SDS-PAGE gel, Western blot was performed using a serum pool of dengue-positive patients, and the PAS reaction confirmed that the NS1DENV1–4 proteins expressed in *Pichia pastoris* were glycosylated (Figure 4). The results of the four labels correspond to a 45 kDa band.

### 3.3. IgM and IgG Indirect ELISA

**Figure 5:**
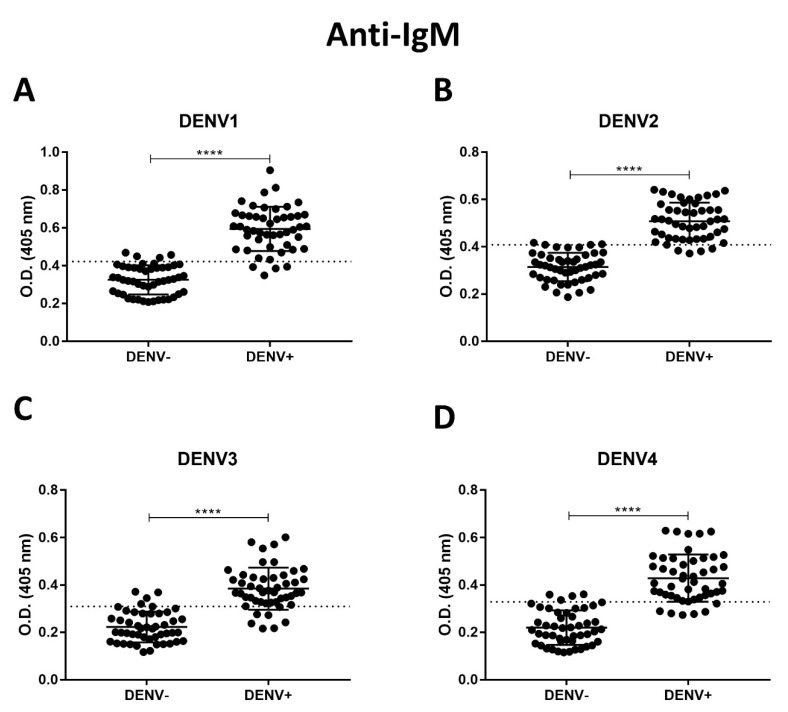
Anti-dengue IgM indirect ELISA, using the recombinant protein as antigen to capture. (**A**) NS1DENV1; (**B**) NS1DENV2; (**C**) NS1DENV3; (**D**) NS1DENV4. (Unpaired *t*-test: **** *p*-value < 0.0001).

**Figure 6:**
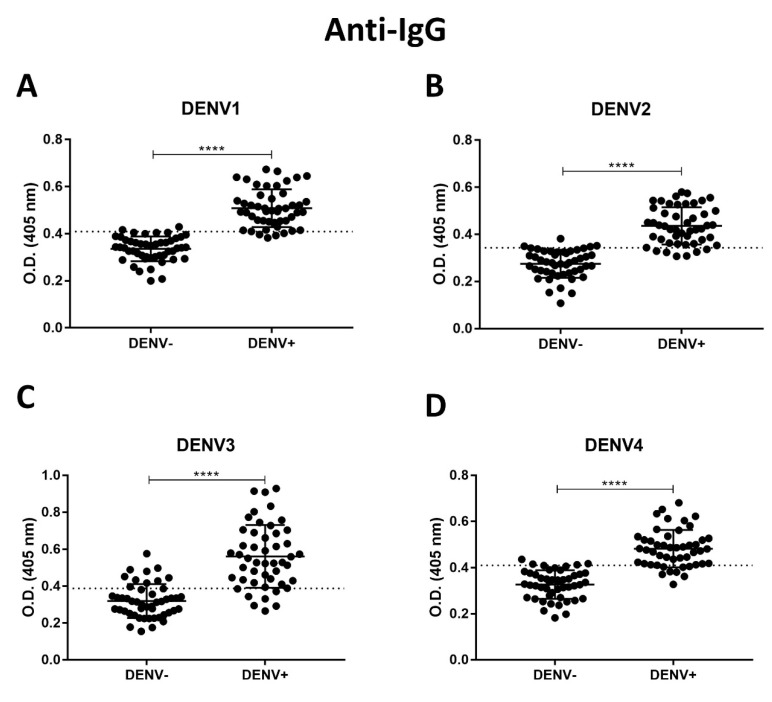
Anti-dengue IgG indirect ELISA, using the recombinant protein as antigen to capture. (**A**) NS1DENV1; (**B**) NS1DENV2; (**C**) NS1DENV3; (**D**) NS1DENV4. (Unpaired *t*-test: **** *p*-value < 0.0001).

Recombinant NS1DENV1–4 proteins were used as the detection antigen in ELISA assays for anti-dengue antibody detection. Results obtained for anti-dengue IgM (Table 2, Figure 5A–D) showed 85–91% sensitivity and 91–93% specificity, and for anti-dengue IgG (Table 3, Figure 6A–D), 83–87% sensitivity and 81–93% specificity were found. Graphs were reproduced from the ROC curve, and the 95% CI (confidence interval) and best cut-off for each assay were defined.

## 4. Discussion

The NS1 protein (the focus of this work) is the most relevant dengue molecular marker in the development of diagnostic methodologies. The study of new molecular diagnostic tests and disease detection are focused on providing better understanding in case management and more effective and faster clinical evaluation of patients in critical regions. The symptoms that characterize dengue are nonspecific and easily confused with other febrile diseases, so the definitive diagnosis requires laboratory confirmation. Disease outbreaks occur in many regions with poor populations and limited conditions of public health care. Therefore, accurate and cost-effective diagnostic tools are essential for the care, surveillance, investigation, and control of outbreaks [54,55].

Dengue viremia detection can be performed in the initial febrile period, from 0 to 7 days after the onset of symptoms, either by virus isolation, PCR detection, or antigen detection. However, in most cases, symptomatic patients only procure medical care in a more advanced stage, making viral detection a non-viable method. The most frequently used diagnostic approach has been anti-dengue IgM detection, which can be detected from 3 to 5 days and which peaks around 12 to 14 days after the onset of symptoms [56,57]. NS1 is a primary and secreted protein in the early stage of infection, and due to this fact, an immune response is built up in the first few days and rises progressively, circulating anti-NS1 IgM antibodies [58,59,60]. 

In this work, we produced the NS1 proteins of the four dengue viruses in *Pichia pastoris* yeast as an inexpensive alternative to producing the antigen as an input to the manufacture of diagnostic kits. In the first step, the genes were optimized for expression in yeast, and the objectives were reached, with results showing that *P. pastoris* is a good heterologous host and worked for the production of NS1DENV1–4 recombinant proteins. Easy to grow and quick to produce, the yeast secreted ready-to-use proteins, with a final yield estimated at 2.8–4.6 milligrams per liter of culture. These values are in agreement with the yield of other works expressing protein in *P. pastoris* [61,62,63,64,65,66,67]. The transformed yeast thus obtained can be used for production in industrial-scale bioreactors, as demonstrated by Bawa and Routledge [68], Rabert and Weinacker [69], Wei and Braun-Galleani [70], Aw and McKay [71], and Liu and Gong [72].

Densitometry, made from the SDS-PAGE results to quantify the expression of the recombinant proteins during induction days, confirmed that the amount of recombinant protein was at its greatest on the sixth day of induction. The recombinant protein concentration in the BMM induction medium increased as a function of time, while the yeast was in the exponential growth stage. From the sixth day onward, yeast enters into a stationary phase (which precedes the phase of death), and as a consequence its metabolism is reduced and the production of recombinant proteins decreases. A Western blot of the four proteins (NS1DENV1–4) was made using anti-dengue positive human serum. The protein identity was revealed by a nitrocellulose membrane immunostaining for each recombinant *P. pastoris* system. Periodic acid-Schiff (PAS) is a staining used to detect polysaccharides, glycoproteins, and glycolipids in histology and pathology. From the Schiff reagent, a staining method was developed to detect isoforms of glycoproteins after electrophoresis by the purple-magenta color in the membrane [73,74]. This procedure corroborated the expectation that NS1DENV1–4 glycoproteins would be glycosylated when expressed in the yeast *Pichia pastoris*. The stained bands were observed at the same time in the SDS-polyacrylamide gel, Western blotting, and PAS. The estimated molecular weight of the proteins, based on molecular markers, was around 45 kDa. The bands stained with anti-dengue antibodies in the Western blot revealed a subtle characteristic that is very common in protein with a glycosylation site; when expressed in a heterologous form, more than one band is revealed. This result suggests that the lower band refers to the non-glycosylated or a reduced glycosylation form of the recombinant protein. This phenomenon was addressed in the recent work by Wang and Rong [75] that characterized glycosylation in *Pichia pastoris*. They demonstrated the presence of two parallel bands of the same protein with and without glycosylation. Falgout and Chanock [76] revealed the existence of the NS1 protein of the dengue virus in more than one way, related to different glycosylation patterns.

As surmised, *P. pastoris* yeast acted on the protein structure with post-translational modifications, since the amino acid chains encoded by the optimized sequences (Figure 1B–E) would present approximately 40 kDa without the addition of glycans. This observation can be directly related to the improved detection of antibodies by the glycosylated recombinant proteins, which, because they are correctly folded, are recognized by antibodies acting against the dengue NS1 protein specific to the conformational epitope, already reported in human biological samples [77,78]. In the 192 samples tested, we obtained 85–91% sensitivity and 91–93% specificity using IgM as a target, and 83–87% sensitivity and 81–93% specificity for anti-dengue IgG (Table 3). Preserved conformational epitopes certainly contributed to the high degree of specificity and sensitivity.

Tests for the detection of anti-dengue antibodies in serum samples are widely used, especially in underdeveloped countries, due to ease of use when compared to other techniques such as viral RNA detection. In primary infections with DENV, the IgM response has higher titers and is more specific than during subsequent infections. In contrast, the IgG titer is higher in repeat infections. Several IgM and IgG ELISA kits are commercially available, with sensitivity ranges of 21–99% and 8–89%, respectively, and specificities varying from 52% to 100% for IgM and from 63% to 100% for IgG, compared to ELISA tests, which are considered the gold standard. In non-endemic regions, IgM-based tests can be used in clinical surveillance with high probability of positive results to indicate recent infections (during the previous 2 to 3 months) [29,79].

## 5. Conclusions

For future investments, this work demonstrated that the use of yeast for antigen production to detect anti-dengue antibodies is a promising alternative which could contribute to the development of a rapid diagnostic test. The recombinant proteins NS1DENV1–4 thus produced are promising candidates for tests due to their high yield, antigenic integrity, and reduced cost for industrial-scale production.

## Figures and Tables

**Table 1:** Yield of recombinant protein production in *P. pastoris*.

	Abs^562 nm^	[ ]^f^ (µg/mL)	Yield (mg/L)
NS1DENV1	0.1867	1795.0	3.590
NS1DENV2	0.1790	1602.5	3.205
NS1DENV3	0.1707	1395.0	2.790
NS1DENV4	0.2076	2317.5	4.635
Standard curve equation: y = 0.0004x + 0.1149; R^2^ = 0.9963; f: final concentration.			

**Table 2:** Sensitivity and specificity of the anti-dengue IgM indirect ELISA.

Anti-IgM	% Sensitivity	% Specificity	*p*-Value
NS1DENV1	91.67	91.67	≤0.0001
NS1DENV2	91.67	93.75	≤0.0001
NS1DENV3	85.42	91.67	≤0.0001
NS1DENV4	87.50	91.67	≤0.0001
Receiver operating characteristic (ROC) curves were analyzed to estimate the diagnostic sensitivity and specificity. Unpaired *t*-test for significance.			

**Table 3:** Sensitivity and specificity of the anti-dengue IgG indirect ELISA.

Anti-IgG	% Sensitivity	% Specificity	*p*-Value
NS1DENV1	85.42	93.75	≤0.0001
NS1DENV2	87.50	91.67	≤0.0001
NS1DENV3	85.42	81.25	≤0.0001
NS1DENV4	83.33	91.67	≤0.0001
Receiver operating characteristic (ROC) curves were analyzed to estimate the diagnostic sensitivity and specificity. Unpaired *t*-test for significance.			
